# We should care more about intracuff pressure: The actual situation in government sector teaching hospital

**DOI:** 10.4103/0019-5049.68374

**Published:** 2010

**Authors:** Lopa Trivedi, Pramila Jha, Narasi Ram Bajiya, DC Tripathi

**Affiliations:** Department of Anaesthesiology, Government Medical College and Sir T. Hospital, Bhavnagar, Gujarat, India

**Keywords:** Aneroid manometer, intra cuff pressure, polyvinyl chloride endotracheal tube, red rubber endotracheal tube

## Abstract

Endotracheal tube (ETT) should have intracuff pressure (ICP) in the range of 20 to 30 cm water (H_2_O). In this observational study, we studied the trend amongst anaesthesiologist in choosing the type of ETT and their ability to assess optimum ICP clinically. After institutional ethics committee approval, we observed 75 patients under general endotracheal anaesthesia in Government Medical College. Anaesthesiologists were blinded to study purpose. The type of ETT used and magnitude of ICP was recorded. ICP was measured using simple aneroid manometer. Once the pressure was measured, it was readjusted to normal range and nitrous oxide was allowed to start. Red rubber tube was used in 18.7% and polyvinyl chloride (PVC) in 81.3% cases. The anaesthesiologists were not able to assess ICP in the recommended range clinically in 100% cases when red rubber ETT was used and in 40% cases when portex ETT was used. Red rubber ETT (reusable) with low-volume high-pressure cuff is still in use, though the trend is shifting towards more of using PVC ETT. Anaesthesiologists were not able to inflate the ETT cuff to the recommended range in spite of their clinical expertise (more than 5 years of teaching experience) in significant number of cases. We recommend the use of simple aneroid manometer for objective monitoring of ICP over subjective assessment, not only in red rubber, but also in PVC ETT.

## INTRODUCTION

Establishing a secured airway via endotracheal intubation is a basic clinical skill and life-saving technique commonly used by anaesthesiologists in and out of operation theatre. It is also used by other clinicians and paramedics in critical care scenario. This procedure, however, can cause complications even long after the endotracheal tube (ETT) is placed passed the vocal cord and secured.

Sore throat and hoarseness,[1–3] tracheal necrosis,[4] rupture,[5,6] stenosis,[7] laryngeal nerve palsy[8] and tracheo-oesophageal fistula[9] are all potential risks when intracuff pressure (ICP) in ETT is excessively high. Wide varieties of ETTs are available in the market. What is the trend in selecting the type of ETT and ability of the anaesthesiologist of assessing the optimum ICP clinically was observed.

It has long been believed, without any evidence based data, that the trained clinicians are capable of determining proper ICP clinically. In this observational study, we tested the hypothesis that the tube cuff is either inadequately or excessively inflated when cuff pressure was not monitored with a manometer.

## METHODS

After institutional ethics committee approval, this prospective observational study was carried out in 75 adult patients of either sex, ASA grade I – III, chosen randomly from different surgical specialties posted for elective surgery under general endotracheal anaesthesia in the Government Medical College. After thorough preanaesthetic evaluation, patients with history of smoking, cough, sore throat, common cold, chronic respiratory disease, anticipated difficult intubation, high risk of aspiration, nasogastric tube in situ and known anatomical laryngotracheal anomaly were excluded from the study.

All patients underwent a prescribed anaesthetic protocol. Induction was accomplished with intravenous bolus of induction agent and paralysis achieved with succinylcholine. Trachea was intubated by anaesthesiologists having more than 5 years of teaching experience in the specialty with 7 to 7.5 mm ID in females and 9 to 9.5 mm ID cuffed ETT in males of red rubber or polyvinyl chloride (PVC) as per their choice. Intubating anaesthesiologists were blinded to the nature of the study. Patients in whom ETT size other than these were used were excluded from the study. Intubating anaesthesiologists were free to inflate the cuff of ETT as per their clinical judgment and assessment. ETT was connected to breathing system, and anaesthesia was maintained with the method of choice of the anaesthesiologist. However, nitrous oxide (N_2_O) was allowed to start only after ICP measurement was complete.

**Figure 1:**
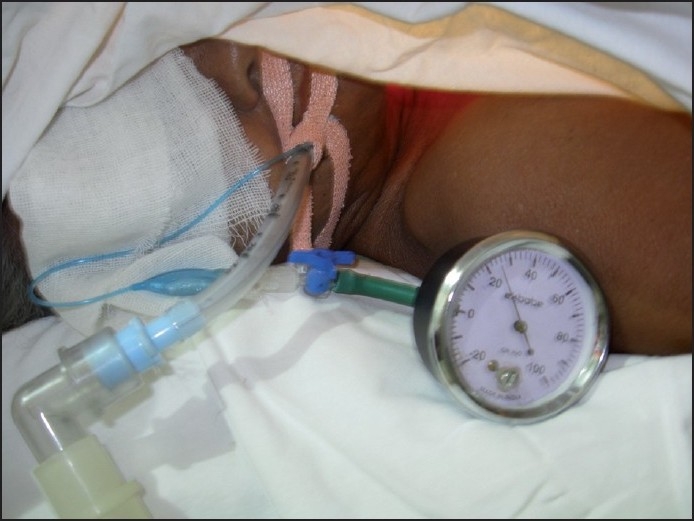
Method of measuring intra cuff pressure

ICP was recorded using simple aneroid manometer (Hospitech India, Bangalore, India) with reading in cm of water (H_2_O) from 0 to 100.Aneroid manometer was connected to the inflating channel of the pilot balloon with a three way stop cock as seen in Figure 1. Once the pressure was measured, it was readjusted to normal range and N_2_O was allowed to start

## Statistical analysis

Our primary outcome was measured ETT cuff pressure. After considering the results of previous studies with average probability of 60% cases falling outside the recommended range of ICP and allowing 20% margin of error, a minimum sample size of 66 is calculated. This study recruited 75 patients, which exceeds minimum sample size required. After permitting 20% margin of error, power of this study stands out to be 80%.

Data were presented as mean ± standard deviation. The percentage of patients with ICP outside the recommended range was also calculated. *P*<0.05 was considered statistically significant. Chi-square test was utilised for testing the hypothesis where appropriate.

## RESULTS

Patient’s characteristics are shown in Table 1. PVC ETT was used in 61 (81.3%) patients, whereas red rubber ETT in 14 (18.7%) patients out of 75 patients observed [Table 2]. ICP was recorded in two types of tubes [Table 3]. Number of patients with ICP within the recommended range and outside the range were calculated [Table 4].

**Table 1:** Demographic data of patients

Demographic profile	Mean ± S.D
Age	31.23 ± 13.15
Sex	
M	48%
F	52%
Weight (Kg)	
M	56.03 ± 9.14
F	50.64 ± 9.78
Height (cm)	
M	162.03 ± 5.98
F	153.28 ± 4.96

**Table 2:** Type of ETT used

Type of ETT	Number of patients	Percentage (%)
PVC	61	81.3
Red rubber	14	18.7

**Table 3:** Mean intra cuff pressure

Type of ETT	Intra cuff pressure (cm H_2_O) Mean ± S.D.
PVC	27.07 ± 10.40
Red rubber	86.0 ± 16.47*

* This is the average of only ten cases as in four cases intra cuff pressure could not be measured. (ICP was beyond the upper limit of aneroid manometer)

**Table 4:** Range of intra cuff pressure

Type of ETT	Intra cuff pressure (cm H_2_O)		
	<20	20 – 30	>30
PVC	9 (14.8)	36 (59.0)	16 (26.2)
Red rubber			14 (100)

Figures in parenthesis are in percentage

## DISCUSSION

Animal data indicate that a cuff pressure of only 20 cm H_2_O may significantly reduce tracheal blood flow with normal blood pressure, and critically reduces it during severe hypotension.[10] Similarly, inflation of ETT cuffs to 20 cm H_2_O for just four hours produces serious ciliary damage that persists for at least three days.[11] One study recommended selecting a cuff pressure of 25 cm of H_2_O as a minimum cuff pressure to prevent aspiration and leaks past the cuff.[12] In another study, the relationship between cuff pressure and capillary perfusion of rabbit tracheal mucosa was studied, and authors recommended that the cuff pressure be kept below 27 cm of H_2_O.[13] Another study recommended that the cuff inflation pressure should not exceed 30 cm of H_2_O.[14] In the absence of clear guidelines, many clinicians consider 20 cm of H_2_O a reasonable lower limit for cuff pressure in adults. It is thus essential to maintain the cuff pressure in the range of 20 to 30 cm of H_2_O. Thus, appropriate inflation of ETT cuff is obviously important.

Even though cuff pressure can be easily measured with a small aneroid manometer,[15] adequacy of cuff inflation was conventionally determined by palpation of pilot balloon[4,16] or by inflating just enough to stop palpable leak around the cuff.

The aim of this study was to observe the trend amongst anaesthesiologists in choosing the type of ETT and their ability to judge ICP adequately by their clinical experience.

In this study of 75 patients, PVC ETT was used in 61 (81.3%) and red rubber in 14 (18.7%) patients. This shows increasing awareness among the anaesthesiologists regarding the risk of over inflation of cuff.

Though, mean ICP was 27.07 ± 10.40 cm of H_2_O in PVC ETT (within normal range), in 14.8% cases, it was below the recommended range and in 26.2%, it was above the recommended range. Cuff pressure, thus in significant number of cases (40%), was outside the recommended range. It is nonetheless encouraging that we observed relatively few extremely high values, at least many fewer than reported in previous studies.[16] These results suggest that clinicians are now making reasonable efforts to avoid grossly excessive cuff inflation. Though the results are encouraging, 40% of cases showing ICP outside the recommended range justifies objective monitoring of ICP in routine practice. Although intubation and cuff inflation were done by experienced anesthesiologists, they were unable to identify optimum ICP in 40% cases by their clinical judgment. It is supported by previous studies that this skill is not acquired over time with increased training or experience.[17,18]

As far as red rubber ETT is concerned, the mean ICP was 86.0 ± 16.47 cm of H_2_O, which is dangerously above the recommended range. Though less in use, red rubber ETT is still being used and certainly carries an inherent risk of airway damage if ICP is not monitored. It is nonetheless a happy scenario that red rubber ETT use is decreasing and it is no more in practice in most of the places. The cost effectiveness in long run may be the only reason for using red rubber tubes in clinical practice.

ICP was recorded before the starting of N_2_O; we can imagine the further increase in ICP after starting N_2_O,[19] as all the anaesthesiologists used air as an inflating agent.

Varieties of newer ETTs are in market now. Lanz tube, with its over-pressure safety balloon, maintained a lateral wall pressure (LWP) below the mean capillary perfusion pressure even when inflated considerably beyond the seal point,[20] and the “super safety yellow” reusable ETT with intermediate volume and low-pressure cuff also prevents excessive build up of ICP.[21] Its performance is comparable to PVC ETT with regard to cuff seal and ICP. Additionally, it helps in reducing PVC waste and may lessen cost in long run. These tubes may be alternative to the currently used disposable tubes in anaesthesia.

The pressure that causes ischaemic damage to the tracheal mucosa is the LWP, not the ICP itself. It has been shown that the two pressures are not equivalent.[22] LWP can be determined by scanning electron microscopy and energy dispersive X-ray analysis, which are though useful and more accurate, are costly techniques.[23]

We conclude that ICP monitoring with simple aneroid manometer is a good and vigilant medical practice even with its short coming (not equal to LWP). We recommend the use of objective monitoring of ICP over subjective assessment not only in red rubber, but also in PVC ETT.

## References

[1] Knowlson GT, Bassett HF (1970). The pressure exerted on the trachea by endotracheal inflatable cuffs. Br J Anesth.

[2] Loeser EA, Orr DL (1976). Endotracheal tube cuff design and postoperative sore throat. Anesthesiology.

[3] Reader JC, Borochgrevink PC, Sellevold OM (1985). Tracheal tube cuff pressure. Anaesthesia.

[4] Fernandez R, Blanch L, Mancebo J, Bonsoms N, Artigas A (1990). Endotracheal tube cuff pressure assessment: Pitfalls of finger estimation and need for objective measurement. Crit Care Med.

[5] Fan CM, Ko PC, Tsai KC, Chiang WC, Chang YC, Chen WJ (2004). Tracheal rupture complicating emergent endotracheal intubation. Am J Emerg Med.

[6] Harris R, Joseph A (2000). Acute tracheal rupture related to endotracheal intubation: Case report. J Emerg Med.

[7] Terashima H, Sakurai T, Takahashi S, Saitoh M, Hirayama K (2002). Postintubation tracheal stenosis: Problems associated with choice of management. Kyobu Geka.

[8] Lu YH, Hsieh MW, Tong YH (1999). Unilateral vocal cord paralysis following endotracheal intubation a case report. Acta Anaesthesiol Sin.

[9] Pelc P, Prigogine T, Bisschop P, Jortay A (2001). Tracheoesophageal fistula: Case report and review of literature. Acta Otorhinolaryngol Belg.

[10] Bunegin L, Albin MS, Smith RB (1993). Canine tracheal blood flow after endotracheal tube cuff inflation during normotension and hypotension. Anesth Analg.

[11] Sanada Y, Kojima Y, Fonkalsrud EW (1982). Injury of cilia induced by tracheal tube cuffs. Surg Gynecol Obstet.

[12] Lomholt N (1992). A device for measuring the lateral wall cuff pressure of endotracheal tubes. Acta Anaesthesiol Scand.

[13] Nordin U, Lindholm CE, Wolgast M (1977). Blood flow in the rabbit tracheal mucosa under normal conditions and under the influence of tracheal intubation. Acta Anaesthesiol Scand.

[14] Seegobin RD, Van Hasselt GL (1984). Endotracheal cuff pressure and tracheal mucosal blood flow: Endoscopic study of effects of four large volume cuffs. Br Med J (Clin Res Ed).

[15] Bouvier JR (1981). Measuring tracheal tube cuff pressures-tool and technique. Heart Lung.

[16] Braz J, Navarro L, Takata I, Nascimento P (1999). Endotracheal tube cuff pressure: Need for precise measurement. Sao Paulo Med J.

[17] Parwani V, Hahn I, Hsu B, Hoffman R (2004). Experienced emergency physicians cannot safely or accurately inflate endotracheal tube cuffs or estimate endotracheal tube cuff pressure using standard technique. Acad Emerg Med.

[18] Parwani V, Hahn I, Hoffman R (2006). Experienced paramedics cannot inflate or estimate endotracheal tube cuff pressure using standard techniques. Ann Emerg Med.

[19] Stanely TH, Kawamura R, Graves C (1974). Effects of nitrous oxide on volume and pressure of endotracheal tube cuffs. Anesthesiology.

[20] Leigh JM, Maynard JP (1979). Lenz tube. Br Med J.

[21] Hähnel J, Treiber H, Konrad F, Mutzbauer T, Steffen P, Georgieff M (1994). Performance characteristic of a novel reusable intermediate volume low pressure cuffed endotracheal tube. Acta Anaesthesiol Scand.

[22] Black AM, Seegobin RD (1981). Pressures on endotracheal tube cuffs. Anaesthesia.

[23] Wu WH, Arteaga M, Mlodozeniec AR (2004). Surface ultra structure and pressure dynamics of tracheal tube cuffs. J Biomed Mater Res.

